# Wildlife as Sentinels of Antimicrobial Resistance in Germany?

**DOI:** 10.3389/fvets.2020.627821

**Published:** 2021-01-27

**Authors:** Carolina Plaza-Rodríguez, Katja Alt, Mirjam Grobbel, Jens Andre Hammerl, Alexandra Irrgang, Istvan Szabo, Kerstin Stingl, Elisabeth Schuh, Lars Wiehle, Beatrice Pfefferkorn, Steffen Naumann, Annemarie Kaesbohrer, Bernd-Alois Tenhagen

**Affiliations:** ^1^Department Biological Safety, German Federal Institute for Risk Assessment (BfR), Berlin, Germany; ^2^Department Food, Feed, Consumer Goods, German Federal Office of Consumer Protection and Food Safety (BVL), Berlin, Germany

**Keywords:** monitoring, one health, zoonotic agents, antimicrobial resistance (AMR), wild boar, cervids, wild bird, Germany

## Abstract

The presence of bacteria carrying antimicrobial resistance (AMR) genes in wildlife is an indicator that resistant bacteria of human or livestock origin are widespread in the environment. In addition, it could represent an additional challenge for human health, since wild animals could act as efficient AMR reservoirs and epidemiological links between human, livestock and natural environments. The aim of this study was to investigate the occurrence and the antibiotic resistance patterns of several bacterial species in certain wild animals in Germany, including wild boars (*Sus scrofa*), roe deer (*Capreolus capreolus*) and wild ducks (family Anatidae, subfamily Anatinae) and geese (family Anatidae, subfamily Anserinae). In the framework of the German National Zoonoses Monitoring Program, samples from hunted wild boars, roe deer and wild ducks and geese were collected nationwide in 2016, 2017, and 2019, respectively. Fecal samples were tested for the presence of *Salmonella* spp. (in wild boars and wild ducks and geese), *Campylobacter* spp. (in roe deer and wild ducks and geese), Shiga toxin-producing *Escherichia* (*E*.) *coli* (STEC), commensal *E. coli* and extended-spectrum beta-lactamase- (ESBL) or ampicillinase class C (AmpC) beta-lactamase-producing *E. coli* (in wild boars, roe deer and wild ducks and geese). In addition, the presence of methicillin-resistant *Staphylococcus aureus* (MRSA) was investigated in nasal swabs from wild boars. Isolates obtained in the accredited regional state laboratories were submitted to the National Reference Laboratories (NRLs) for confirmation, characterization and phenotypic resistance testing using broth microdilution according to CLSI. AMR was assessed according to epidemiological cut-offs provided by EUCAST. *Salmonella* spp. were isolated from 13 of 552 (2.4%) tested wild boar fecal samples, but absent in all 101 samples from wild ducks and geese. Nine of the 11 isolates that were submitted to the NRL Salmonella were susceptible to all tested antimicrobial substances. *Campylobacter* spp. were isolated from four out of 504 (0.8%) roe deer fecal samples, but not from any of the samples from wild ducks and geese. Of the two isolates received in the NRL *Campylobacter*, neither showed resistance to any of the substances tested. From roe deer, 40.2% of the fecal samples (144 of 358) yielded STEC compared to 6.9% (37 of 536) from wild boars. In wild ducks and geese, no STEC isolates were found. Of 150 STEC isolates received in the NRL (24 from wild boars and 126 from roe deer), only one from each animal species showed resistance. Of the 219 isolates of commensal *E. coli* from wild boars tested for AMR, 210 were susceptible to all 14 tested substances (95.9%). In roe deer this proportion was even higher (263 of 269, 97.8%), whereas in wild ducks and geese this proportion was lower (41 of 49, 83.7%). Nevertheless, selective isolation of ESBL-/AmpC-producing *E. coli* yielded 6.5% (36 of 551) positive samples from wild boars, 2.3% (13 of 573) from roe deer and 9.8% (10 of 102) from wild ducks and geese. Among the 25 confirmed ESBL-/AmpC-producing isolates from wild boars, 14 (56.0%) showed resistance up to five classes of substances. This proportion was lower in roe deer (3 of 12, 25%) and higher in wild ducks and geese (7 of 10, 70%). None of the 577 nasal swabs from wild boars yielded MRSA. Results indicate that overall, the prevalence of resistant bacteria from certain wild animals in Germany is low, which may reflect not only the low level of exposure to antimicrobials but also the low level of resistant bacteria in the areas where these animals live and feed. However, despite this low prevalence, the patterns observed in bacteria from the wild animals included in this study are an indicator for specific resistance traits in the environment, including those to highest priority substances such as 3rd generation cephalosporins, fluoroquinolones and colistin. Therefore, also continuous monitoring of the occurrence of such bacteria in wildlife by selective isolation is advisable. Furthermore, the possible role of wildlife as reservoir and disperser of resistant bacteria would need to be assessed, as wild animals, and in particular wild ducks and geese could become spreaders of resistant bacteria given their capacity for long-range movements.

## Introduction

The presence of bacteria carrying antimicrobial resistance (AMR) genes is an increasingly serious and complex threat affecting public health worldwide (1). This implies that all underlying economic, social, political, environmental, and biological factors have to be considered in this context (2). Nowadays intensive contact between humans, domestic and wild animals occurs due to the expansion of urban populations and the fragmentation, encroachment and loss of natural habitats. In this scenario, it is of utmost importance to examine AMR through a “One Health” perspective (3–5). This perspective contemplates an integrated and holistic multidisciplinary approach (6), highlighting the importance of a better integration of human, livestock, wildlife and environmental aspects, in order to identify key priorities for combating AMR (2, 5, 7).

Even though wild animals are unlikely of being treated with antibiotics, the overlap between habitats inevitably increases the transmission of resistant bacteria between the different niches (8). Some wild species have been used as bioindicators or sentinels for the spread of resistant bacteria in the environment (9–11). Inadequately treated waste from humans and livestock animals treated with antimicrobial substances promotes the spread of resistant bacteria from animal stables and waste water treatment plants to the environment (12–14), and therefore to the wild fauna. However, despite the fact that many studies affirm that wild animals are reservoirs and dispersers of AMR, this role is less well-established. To make this statement, more in-depth epidemiological analyzes are needed, as the mere fact of being carriers of AMR does not mean that they can be a vehicle of contagion for humans or other animals (15, 16). In consequence, it becomes important to study the presence of AMR genes in wildlife and consider the role of wild animals in the dynamics of AMR (15), as they could represent a major epidemiological link between natural and humanized environments (15, 16). Roe deer (*Capreolus capreolus*) and wild boar (*Sus scrofa*) are the most frequent and widespread wild ungulates in Germany (17), with an estimated number of around 2.4 million individuals of roe deer and one million of wild boars, which represents 24 and 25% of the total European wild boar and roe deer population, respectively (18). As an ecologically adaptable species, both can be found in a wide variety of habitats from natural ones like forests or pastures, to more anthropogenic areas like agricultural landscapes and even urban or peri-urban areas (18, 19). Therefore, they might be prone to have contact to humans and livestock directly (20), as well as indirectly via garbage and sewage. On the other hand, some wild bird populations, including wild ducks and geese belonging to different species within the Anatidae family and the Anatinae and Anserinae subfamilies, have experienced extraordinary growth in the last decades in Germany (21, 22). Among other reasons, this is due to milder winter conditions (21). It is therefore not unusual nowadays to find large groups of wild ducks and geese in crops producing food and feed, or on wetlands and lakes used as source of drinking water for humans and livestock, or for aquatics (23, 24). Due to their capacity for long-range movements, wild birds like ducks and geese are potential spreaders of bacteria with AMR genes beyond borders (16, 25–27).

Previous studies have demonstrated the presence of AMR and resistance genes in bacteria from a large variety of wildlife species throughout Europe (28–30), including resistances to those substances of highest priority like 3rd generation cephalosporins, fluoroquinolones, colistin or even carbapenems (31, 32).

To the best of our knowledge, in Germany, the availability of studies regarding the presence of resistant bacteria in wild animals is scarce and mostly limited to certain regions (33–35). This makes that the role of wild animals in the dynamics of AMR in Germany is still not fully understood. Based on previous studies it is clear that the presence of distinct bacterial species, their antimicrobial susceptibility, as well as their profiles of resistance genes might be highly variable among different countries (19). Therefore, the aim of the present study was to investigate the occurrence and the antibiotic resistance patterns of *Salmonella* spp., *Campylobacter* spp., Shiga toxin-producing *Escherichia* (*E*.) *coli* (STEC), methicillin-resistant *Staphylococcus aureus* (MRSA), commensal *E. coli*, and extended-spectrum beta-lactamase- (ESBL) or ampicillin class C (AmpC) beta-lactamase-producing *E. coli* in samples collected from wild boars, roe deer and wild ducks and geese in Germany within the National Zoonoses Monitoring Program.

## Materials and Methods

In the framework of the German National Zoonoses Monitoring Program, 942 samples from hunted wild boars, 573 from roe deer and 100 from wild ducks and geese were collected nationwide in 2016, 2017, and 2019, respectively. Samples from wild ducks and geese mainly originated from cadavers collected for the monitoring of avian influenza, or taken from hunted birds. Fecal samples were tested for the presence of *Salmonella* spp. (in wild boars and wild ducks and geese), *Campylobacter* spp. (in roe deer and wild ducks and geese), STEC, commensal *E. coli*, and ESBL-/AmpC-producing *E. coli* (in wild boars, roe deer and wild ducks and geese) (Table 1). In addition, the presence of MRSA was investigated in nasal swabs from wild boars. No sample size was specified for each federal state, as the investigations took place depending on the availability of suitable samples. Samples were provided from all federal states except Hamburg and Bremen.

**Table 1:** Overview of prevalence and resistance studies carried out for wildlife in the German Zoonoses-Monitoring in 2016, 2017, and 2019.

**Year**	**Animal**	**Matrix**	***Salmonella* spp**.	***Campylobacter* spp**.	**STEC**	**MRSA**	**Commensal *E. coli***	**ESBL-/ AmpC-producing *E. coli***
2016	Wild boar	Feces	X		X		X	X
2016	Wild boar	Nasal swabs				X		
2017	Roe deer	Feces		X	X		X	X
2019	Wild ducks and geese	Feces	X	X	X		X	X

Primary isolation was carried out by the accredited regional state laboratories using harmonized procedures (Table 2). Results of the analysis of samples were reported to the Federal Office of Consumer Protection and Food Safety (BVL) for aggregation and reporting at national level. Isolates obtained were submitted to the National Reference Laboratories (NRLs) at the German Federal Institute for Risk Assessment (BfR) for confirmation, characterization and phenotypic resistance testing.

**Table 2:** Microbiological methods used in the investigation according to microorganism and survey year.

**Microorganism**	**Year**	**Primary isolation**	**Confirmation and further typing**
*Salmonella* spp.	2016 2019	ISO 6579:2002 ISO 6579-1:2017	ISO 6579:2002 ISO 6579-1:2017 Serotyping according to the White-Kauffmann-Le Minor scheme (36)
*Campylobacter* spp.	2017 2019	ISO 10272-1:2006 ISO 10272-1:2017	ASU §64 LFGB, L00.06-32 2013-08
STEC	2016 2017 2019	Suggested methods dependent on the matrix: - ISO/TS 13136:2012 and ISO based method in 2016 - ASU §64 LFGB, L00.00-92 2006-12 - ASU §64 LFGB L07.18-1 2002-05 - Real-time PCR systems for the detection of the Shiga toxin genes *stx1* and *stx2* and the intimin gene eae in 2016 and 2017	Confirmation and typing for virulence genes as described by Tzschoppe et al. (37). Molecular H-typing according to Beutin et al. (38). Verotoxin ELISA (RIDASCREEN Verotoxin enzyme immunoassay #C2201, R-biopharm, Germany) according to the manufacturer
	2019	Suggested methods: - DIN 10118 “Microbiological examination of food—Detection of verotoxins in food of animal origin with an immunological test system” - Protocol for the qualitative detection and isolation of shigatoxin-producing *E. coli* (STEC) - Detection of *E. coli* producing the Stx2f subtype by Real-Time PCR (EU-RL VTEC: Laboratory methods for VTEC detection and typing (https://www.iss.it/documents/20126/1049000/EU_RL_VTEC_Method_10_Rev_0.pdf)	
MRSA	2016 2017 2019	Recommended method of the National Reference Laboratory for staphylococci including *S. aureus* at the Federal Institute for Risk Assessment (39)	In-house multiplex PCR test (39) and broth microdilution method according to CLSI M07-A10 and classification according to EFSA (40)
ESBL-/AmpC-producing *E. coli*	2016 2017 2019	EURL laboratory protocol for the Isolation of ESBL-, AmpC-, and carbapenemase-producing *E. coli* from caecal samples Version 3 in 2016 and 2017 EURL laboratory protocol for the Isolation of ESBL-, AmpC-, and carbapenemase-producing *E. coli* from caecal samples Version 6	Broth microdilution method according to CLSI M07-A10 and classification according to 2013/652/EU and EFSA (41)
Commensal *E. coli*	2016 2017 2019	No specific standardized method is prescribed. It is just recommended to plate a small amount of feces directly on a suitable medium. Confirmation with in-house method.	Cultivation on ENDO-Agar (Thermo Scientific, Germany)

Isolates from *Salmonella* spp., *Campylobacter* spp., STEC, *E. coli*, and MRSA were confirmed and characterized using the designated, internationally recognized procedures (Table 2). For the determination of resistance, broth microdilution method according to CLSI M07-A10 and CLSI M45-A was used (42, 43).

The isolates were subjected to the examination spectrum of antimicrobial substances established at BfR. For this purpose, the ready-made plate formats EUVSEC and EUVSEC2 (*Salmonella* spp. and *E. coli*), EUCAMP2 (*Campylobacter* spp.), and EUST (MRSA) from the company TREK Diagnostic Systems were used (44).

AMR was assessed according to epidemiological cut-offs provided by the European Committee on Antimicrobial Susceptibility Testing (EUCAST) and fixed in Commission Implementing Decision 2013/652/EC (45). Technical specifications proposed by EFSA (40) were applied for MRSA.When no epidemiological cut-off values were described, the evaluation was carried out based on EFSA criteria (41). Isolates from the wild-type population in this publication are further called susceptible to the respective agent, those with MIC values above the cut-off resistant. An overview of the antimicrobial substances used, the tested concentration ranges as well as the evaluation criteria can be found in Supplementary Tables 1–3.

Prevalence of the zoonotic pathogens in the fecal samples from wild animals as well as the prevalence of resistant bacteria within the isolates were calculated as the proportion of positive samples resp. resistant isolates and with the associated 95% confidence interval shown. The 95% confidence interval was calculated according to the procedure determined by Agresti and Coull (46).

*Escherichia coli* isolates resistant to third generation cephalosporins were further characterized in regard of the harbored ESBL/pAmpC genes. Therefore, isolates were pre-screened by real-time PCR for the presence of the typical betalactamases TEM, CTX, SHV, and CMY (47). ESBL variant was then determined by Sanger sequencing of PCR products. TEM variant was only determined in case no other ESBL/pAmpC gene was detected, as most *E. coli* harbor the narrow spectrum beta-lactamase *bla*_TEM−1_. Isolates which were negative in real-time PCR were additionally screened for the presence of *bla*_FOX_, *bla*_MOX_, *bla*_CIT_, *bla*_DHA_, and *bla*_EBC_ by PCR. As some betalactamase variants differ within the primer regions, we could not distinguish between CTX-M-14 and −17 (CTX-M-14 like), between CTX-M-65 and 90 (CTX-M-65-like), and between CMY-2/-22 and -66 (CMY-2-like).

## Results

### *Salmonella* spp.

*Salmonella* spp. were isolated from 13 of 552 (2.4%) wild boar fecal samples (Table 3).

**Table 3:** Overview of the examined samples and the prevalence and 95% confidence interval (95% CI) of different microorganisms in feces samples (*Salmonella* spp., *Campylobacter* spp., STEC, commensal *E. coli*, and ESBL-/AmpC-producing *E. coli*) and nasal swabs (MRSA) from wild boar, roe deer and wild ducks and geese in 2016, 2017 and 2019, respectively.

	**Wild boars (2016)**			**Roe deer (2017)**			**Wild ducks and geese (2019)**		
	**Examined samples**	**Positive samples**	**Prevalence (in %) (95% CI)**	**Examined samples**	**Positive samples**	**Prevalence (in %) (95% CI)**	**Examined samples**	**Positive samples**	**Prevalence (in %) (95% CI)**
*Salmonella* spp.	552	13	2.4 (1.3–4.0)				101	0	0.0 (0.0–4.4)
*Campylobacter* spp.				504	4	0.8 (0.2–2.1)	93	0	0.0 (0.0–4.8)
STEC	536	37	6.9 (5.0–9.4)	358	144	40.2 (35.3–45.4)	95	0	0.0 (0.0–4.7)
MRSA	577	5^*^	0.0 (0.0–0.8)						
Commensal *E. coli*	538	511	95.0 (92.8–96.6)	573	537	93.7 (91.4–95.4)	102	51	50.0 (40.5–59.5)
ESBL-/AmpC-producing *E. coli*	551	36	6.5 (4.7–8.9)	573	13	2.3 (1.3–3.9)	102	10	9.8 (5.2–17.3)
Total	942			573			100		

* *Isolates not confirmed in the reference laboratory*.

Of the 13 isolates found in fecal samples from wild boars, 11 were submitted to the BfR. Serotyping of these isolates resulted in three *Salmonella* Enteritidis, one *Salmonella* Typhimurium, one *Salmonella* Stanleyville, and six *Salmonella enterica* subspecies I., that could not be further identified by serotyping.

**Figure 1:**
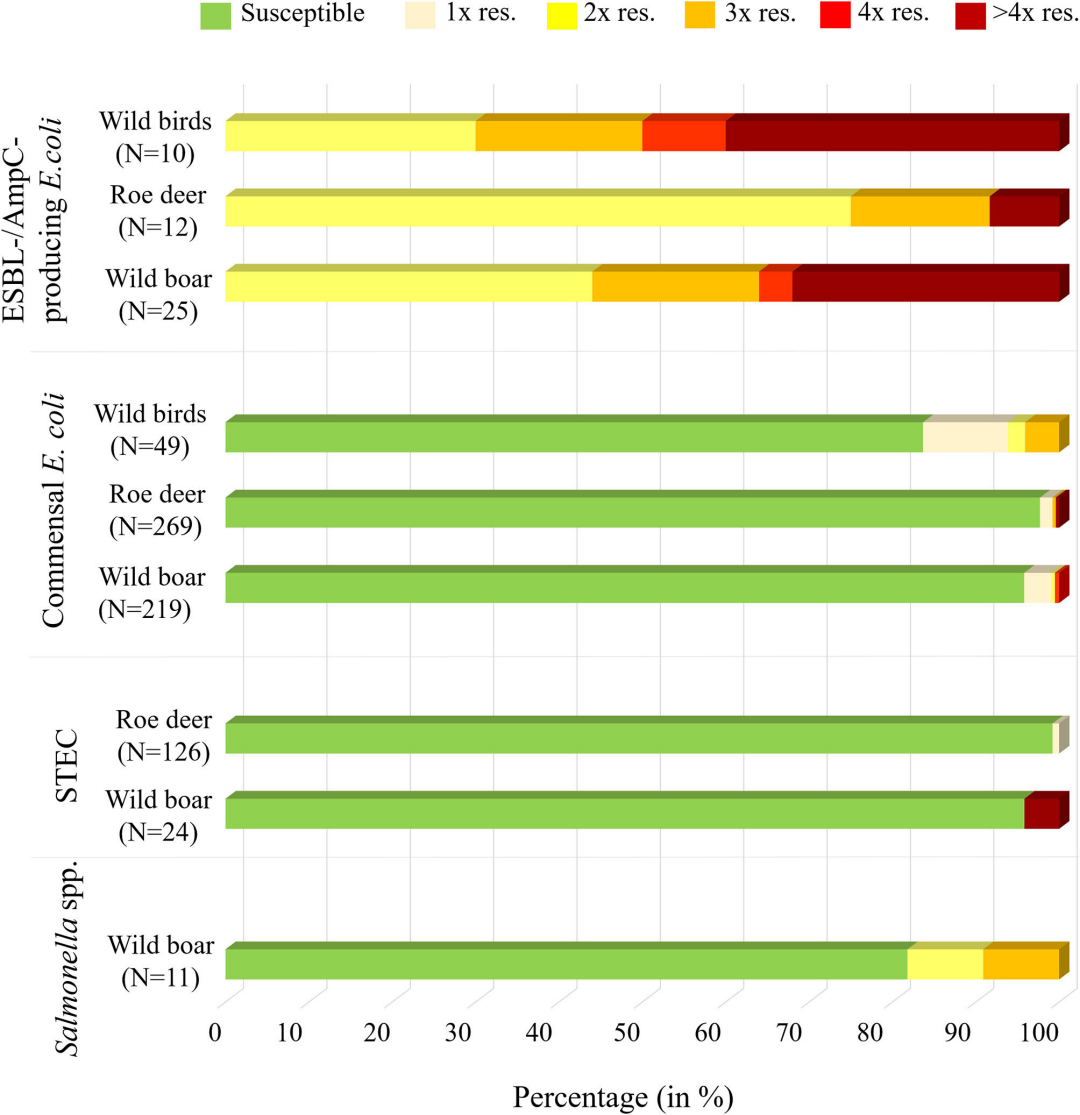
Overview of the isolates found in wild boar, roe deer (excluding *Campylobacter* spp.) and wild ducks and geese, including information on the percentage of samples that were susceptible to al testes substances or resistant to one (1x res.), two (2x res.), three (3x res.), four (4x res.) or more than four classes (> 4x res.) of antibiotic substances.

Of the 11 isolates, nine (81.8%) were susceptible to all tested substances (Table 4). Just two isolates (18.2%) showed resistance to two or three groups of active ingredients (Figure 1), including fluoroquinolones and colistin (Table 5).

**Table 4:** Overview of the isolates for which a resistance test was carried out and prevalence and 95% confidence interval (95% CI) of resistant isolates.

	**Wild boars (2016)**			**Roe deer (2017)**			**Wild ducks and geese (2019)**		
	**Total isolates**	**Resistant isolates**	**Prevalence (in %) (95% CI)**	**Total isolates**	**Resistant isolates**	**Prevalence (in %) (95% CI)**	**Total isolates**	**Resistant isolates**	**Prevalence (in %) (95% CI)**
*Salmonella* spp.	11	2	18.2 (4.0–48.8)				0		
*Campylobacter* spp.				2	0	0.0 (0.0–71.0)	0		
STEC	24	1	4.2 (0.0–21.9)	126	1	0.8 (0.0–4.8)	0		
MRSA	0								
Commensal *E. coli*	219	9	4.1 (2.1–7.7)	269	6	2.2 (0.9–4.9)	49	8	16.3 (8.2–29.3)
ESBL-/AmpC-producing *E. coli*	25	25	100.0 (84.2–100.0)	12	12	100.0 (71.8–100.0)	10	10	100.0 (67.9–100.0)

**Table 5:** Number and proportion of tested resistant isolates from wild boars and the number of substance classes to which the isolates were resistant.

	***Salmonella*** **spp**.		**STEC**		**Commensal** ***E. coli***		**ESBL-/ AmpC-producing** ***E. coli***	
	**N**	**%**	**N**	**%**	**N**	**%**	**N**	**%**
**No. samples**	**11**		**24**		**219**		**25**	
Gentamicin	0	0.0	1.0	4.2	0	0.0	5	20.0
Chloramphenicol	1	9.1	1.0	4.2	2	0.9	4	16.0
Cefotaxime	0	0.0	0.0	0.0	0	0.0	25	100
Ceftazidime	0	0.0	0.0	0.0	0	0.0	24	96.0
Nalidixic acid	1	9.1	1.0	4.2	2	0.9	4	16.0
Ciprofloxacin	1	9.1	1.0	4.2	2	0.9	8	32.0
Ampicillin	1	9.1	1.0	4.2	1	0.5	25	100
Colistin	1	9.1	0.0	0.0	4	1.8	0	0.0
Sulfamethoxazole	0	0.0	1.0	4.2	2	0.9	7	28.0
Trimethoprim	0	0.0	1.0	4.2	2	0.9	6	24.0
Tetracycline	1	9.1	1.0	4.2	1	0.5	9	36.0
Azithromycin	0	0.0	0.0	0.0	0	0.0	2	8.0
Meropenem	0	0.0	0.0	0.0	0	0.0	0	0.0
Tigecycline	0	0.0	0.0	0.0	0	0.0	0	0.0
Susceptible	9	81.8	23.0	95.8	210	95.9	0	0.0
1x resistant	0	0.0	0.0	0.0	7	3.2	0	0.0
2x resistant	1	9.1	0.0	0.0	1	0.5	11	44.0
3x resistant	1	9.1	0.0	0.0	0	0.0	5	20.0
4x resistant	0	0.0	0.0	0.0	1	0.5	1	4.0
>4x resistant	0	0.0	1.0	4.2	0	0.0	8	32.0

*Salmonella* spp. were not found in any of the 101 samples from wild ducks and geese (Table 3).

### *Campylobacter* spp.

*Campylobacter* spp. were isolated from four out of 504 (0.8%) fecal samples from hunted roe deer (Table 3). Three isolates were sent to the BfR, but one of them could not be re-cultivated. Of the two remaining isolates (both *Campylobacter jejuni*), neither showed resistance to any of the six substances tested (Table 4).

*Campylobacter* spp. were absent in the 93 fecal samples from wild ducks and geese (Table 3).

### STEC

Out of 536 fecal samples tested from wild boars, 37 yielded STEC (6.9%) (Table 3). In total, 24 STEC isolates were sent to the BfR for further typing and resistance testing. The results of the STEC typing from wild boars are available in Supplementary Table 4. From those isolates, three did not produce measurable Shiga toxin. With the exception of one isolate, all isolates had a *stx2* gene; meanwhile just five isolates carried a *stx1* gene. One isolate could not be typed with regard to its O antigen, but was serologically rough. The rest of the isolates belonged to 14 different O groups, including the O157 group. The two isolates belonging to this group had both also the H7 antigen and the genes *eae* and *ehxA*, which code for virulence factors. The *eae* gene was also detected in isolates from serogroups O26 and O45. These isolates also carried the *ehxA* gene. The *eae* gene was not found in any other serogroup. The *ehxA* gene was detected in 15 isolates (62.5%).

From the 24 STEC isolates from wild boars tested for resistance, all were completely susceptible except one (95.8%). This isolate showed resistance to six substance classes (Figure 1), including the (fluoro-)quinolone nalidixic acid and ciprofloxacin (Table 5).

From roe deer, 40.2% (144 of 358) of the fecal samples yielded STEC. One hundred twenty-six STEC isolates from the feces of hunted deer were submitted to the BfR. The results of the STEC typing from roe deer are available in Supplementary Table 5. Twenty-five of these 126 isolates did not produce measurable Shiga toxin with the ELISA system used. Most of the isolates had a *stx2* gene (*n* = 92) and 40 isolates carried a *stx1* gene. One hundred fifteen isolates belonged to 19 different O serogroups, and 11 could not be typed. Of the serogroups, O146 was most frequently represented, meanwhile the serogroup O157 was not detected in any of the analyzed isolates. The *eae* gene occurred in one isolate of the serogroup O26. This isolate also carried the *ehxA* gene. The *ehxA* gene was detected in 54 isolates.

Of the 126 STEC isolates tested for resistance, only one (0.8%) showed resistance to gentamicin. As shown in Figure 1 and Table 6, all the other isolates were without exception susceptible to all tested substances.

**Table 6:** Number and proportion of tested resistant isolates from roe deer and the number of substance classes to which the isolates were resistant.

	**STEC**		**Commensal** ***E. coli***		**ESBL-/ AmpC-producing** ***E. coli***	
	**N**	**%**	**N**	**%**	**N**	**%**
**No. samples**	**126**		**269**		**12**	
Gentamicin	1	0.8	0	0.0	1	8.3
Chloramphenicol	0	0.0	0	0.0	0	0.0
Cefotaxime	0	0.0	1	0.4	12	100.0
Ceftazidime	0	0.0	1	0.4	12	100.0
Nalidixic acid	0	0.0	0	0.0	0	0.0
Ciprofloxacin	0	0.0	1	0.4	1	8.3
Ampicillin	0	0.0	4	1.5	12	100.0
Colistin	0	0.0	0	0.0	0	0.0
Sulfamethoxazole	0	0.0	3	1.1	2	16.7
Trimethoprim	0	0.0	2	0.7	2	16.7
Tetracycline	0	0.0	2	0.7	1	8.3
Azithromycin	0	0.0	1	0.4	0	0.0
Meropenem	0	0.0	0	0.0	0	0.0
Tigecycline	0	0.0	0	0.0	0	0.0
Susceptible	125	99.2	263	97.8	0	0.0
1x resistant	1	0.8	4	1.5	0	0.0
2x resistant	0	0.0	0	0.0	9	75.0
3x resistant	0	0.0	1	0.4	2	16.7
4x resistant	0	0.0	0	0.0	0	0.0
>4x resistant	0	0.0	1	0.4	1	8.3

In wild ducks and geese, no STEC isolates were found.

### Commensal *E. coli*

Commensal *E. coli* were isolated from 95% (511 of 538) of the fecal samples from wild boars. Of the 219 isolates of *E. coli* from wild boars tested for AMR, 210 (95.9%) were susceptible to all 14 tested substances (Table 4). Among the nine other isolates, seven showed resistance only to one substance class. The other two isolates showed resistance to two or four classes (Figure 1). No resistance to 3rd generation cephalosporins or carbapenems was found, but some isolates were resistant to ciprofloxacin and nalidixic acid (0.9% each) and four isolates (1.8%) showed resistance to colistin (Table 5).

A total of 93.7% (537 of 573) of the fecal samples from hunted roe deer yielded commensal *E. coli*. Among the 269 isolates, 263 (97.8%) were susceptible to all tested substances, while six (2.2%) displayed resistance to at least one of the tested antimicrobials (Table 4). Four of these isolates were resistant to only one substance class and two isolates were resistant to three, resp. five substance classes (Figure 1). Resistance to the 3rd generation cephalosporins (cefotaxime and ceftazidime) and to the fluoroquinolone ciprofloxacin were observed (0.4% of the isolates each) (Table 6). No colistin or meropenem resistant *E. coli* were observed in isolates from roe deer.

In wild ducks and geese, 50% (51 of 102) of the fecal samples yielded commensal *E. coli*. Of the 49 isolates submitted to the BfR, 41 (83.7%) were sensitive to all tested substances (Table 7). Only two isolates (4.1%) were resistant to two resp. three substance classes (Figure 1). Among the resistant isolates, resistance to 3rd generation cephalosporins, fluoroquinolones and colistin was observed in 2% of the isolates each (Table 7).

**Table 7:** Number and proportion of tested resistant isolates from wild ducks and geese and the number of substance classes to which the isolates were resistant.

	**Commensal** ***E. coli***		**ESBL-/AmpC-producing** ***E. coli***	
	**N**	**%**	**N**	**%**
**No. samples**	**49**		**10**	
Gentamicin	1	2.0	2	20.0
Chloramphenicol	0	0.0	0	0.0
Cefotaxime	1	2.0	10	100.0
Ceftazidime	1	2.0	10	100.0
Nalidixic acid	1	2.0	5	50.0
Ciprofloxacin	1	2.0	5	50.0
Ampicillin	5	10.2	10	100.0
Colistin	1	2.0	0	0.0
Sulfamethoxazole	3	6.1	6	60.0
Trimethoprim	1	2.0	2	20.0
Tetracycline	1	2.0	2	20.0
Azithromycin	0	0.0	1	10.0
Meropenem	0	0.0	0	0.0
Tigecycline	0	0.0	0	0.0
Susceptible	41	83.7	0	0.0
1x resistant	5	10.2	0	0.0
2x resistant	1	2.0	3	30.0
3x resistant	2	4.1	2	20.0
4x resistant	0	0.0	1	10.0
>4x resistant	0	0.0	4	40.0

### ESBL-/AmpC-Producing *E. coli*

**Figure 2:**
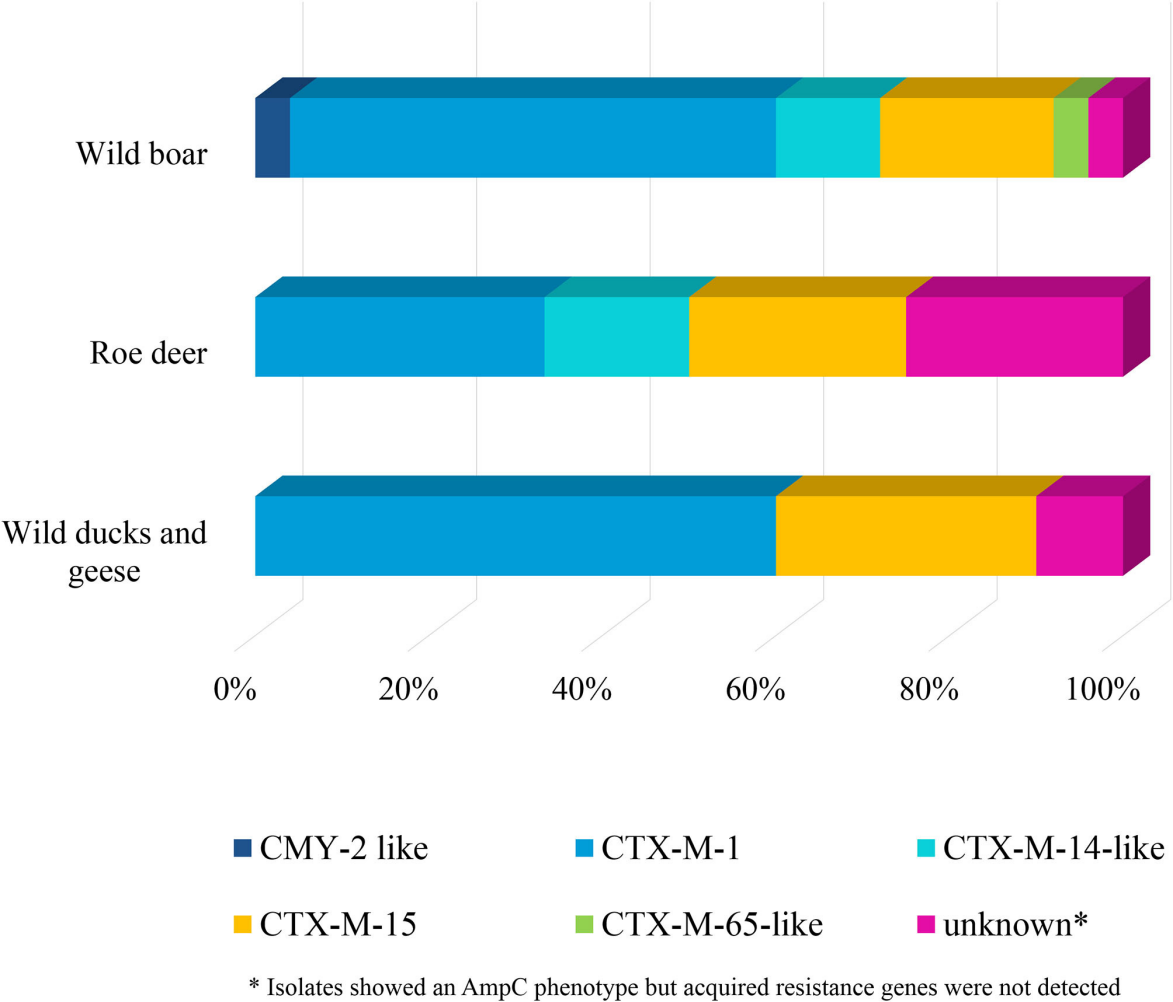
Resistance determinants of *E. coli* isolates obtained from the selective ESBL/AmpC monitoring of wild boars, roe deer, and wild ducks and geese.

Selective isolation yielded isolates suspicious of being ESBL-/AmpC-producing *E. coli* in 6.5% (36 of 551) of the samples from wild boars (Table 3). Of the 25 isolates confirmed at the BfR, 23 showed an ESBL and two an AmpC phenotype. Among these isolates, that were resistant to cefotaxime, ceftazidime and ampicillin, 11 (44%) showed no other resistance, while 14 (56%) showed resistance to up to five further substance classes (Figure 1 and Table 5). Apart from colistin, tigecycline and meropenem, resistance was observed to all other substances in at least one isolate. Nine isolates (36%) were resistant to tetracycline and eight isolates (32%) to ciprofloxacin. Wild boars showed the highest diversity of resistance determinants among the three groups (Figure 2). The most prevalent ESBL gene was *bla*_CTX−M−1_ (56%), followed by *bla*_CTX−M−15_ (20%) and *bla*_CTX−M−14_-like (12%). One of the isolates with an AmpC phenotype harbored a *bla*_CMY−2_ like gene, whereas the other one did not harbor one of the genes screened for.

ESBL-/AmpC-producing *E. coli* were detected in 13 of the 573 (2.3%) fecal samples from hunted roe deer (Table 3). Of the twelve isolates submitted to the BfR, phenotypically three showed an AmpC and nine and ESBL phenotype. Nine of those twelve isolates (75%) showed only resistance to cefotaxime, ceftazidime and ampicillin. Three isolates (25%) showed additional resistances to trimethoprim, sulfamethoxazole, gentamicin, ciprofloxacin or tetracycline (Figure 1 and Table 6). No resistance was observed to chloramphenicol, colistin, meropenem and tigecycline. There was a similar distribution of isolates harboring the ESBL genes *bla*_CTX−M−1_ (33%) and *bla*_CTX−M−15_ (25%) and AmpC-producing isolates (25%). As none of the most prevalent pAmpC genes could be detected, an overexpression of chromosomal AmpC was assumed but not further characterized. The remaining two isolates harbored the *bla*_CTX−M−14_ gene.

In samples from wild ducks and geese, ESBL-/AmpC-producing *E. coli* were isolated from ten of the 102 (9.8%) fecal samples (Table 3). Of the ten isolates submitted to the BfR, eight showed an ESBL phenotype, one an AmpC phenotype, while another one exhibited ESBL and AmpC phenotype. Among the ten confirmed ESBL-/AmpC-producing *E. coli* isolates submitted to the BfR with resistance to cefotaxime, ceftazidime and ampicillin, seven (70%) showed additional resistance up to five classes of substances, including nalidixic acid and ciprofloxacin (in 50% of isolates each). Resistance to colistin or meropenem was not observed (Table 7). The most prevalent ESBL was again CTX-M-1 (60%). CTX-M-15 was produced by 30% of the isolates, including the one which showed an ESBL and AmpC phenotype and produced an additional DHA betalactamase. The isolate with the AmpC phenotype alone only harbored a *bla*_TEM−1_, indicating an additional resistance mechanism which wasn't detected so far.

### MRSA

From the 577 nasal swab samples from wild boars tested, five isolates were found suspicious of being MRSA. However, none of them could be confirmed as MRSA at the BfR (Table 3).

## Discussion

The examination of the fecal samples from wild animals included in this study revealed low levels of the important zoonotic pathogens *Salmonella* spp., *Campylobacter* spp., and MRSA. In contrast, STEC were frequently found in roe deer (40.2%), but infrequently in wild boars and were absent in wild ducks and geese. The antibiotic resistance patterns found in this study indicate that overall, the prevalence of AMR is low in bacteria from the studied wild animals in Germany. This might reflect not only the low level of exposure of these wildlife species to antimicrobials but also the low level of resistant bacteria in the areas where these animals live and feed (16). These good results could be also interpreted as an indication of the low level of anthropogenic impact in these areas, or of an adequate management of antibiotic residues of human or livestock origin in Germany. However, this interpretation should be done with caution, since this study has also shown that wild boars, roe deer and wild ducks and geese are carriers of bacteria with specific resistance traits including colistin, fluoroquinolones or 3rd generation cephalosporins. These substances are considered highest priority critically important antimicrobials by the World Health Organization (48). The origin of these isolates is not known, but due to the lifestyle of the wild animals tested, uptake of the resistant bacteria via feed or drinking water, or through direct contact with garbage and sewages, is a likely reason for carriage (49). Other factors than geographic distance to humans, livestock or wastes should be considered in future studies (50), as it has been demonstrated that wildlife populations living in remote places with little direct human or livestock contact can also harbor resistant bacteria (51). The possible role of wildlife as reservoir and disperser of resistant bacteria in Germany would need to be further assessed by including adequate epidemiological analysis, as wild animals, and in particular, wild ducks and geese could become spreaders of resistant bacteria given their capacity for long-range movements. Samples included in this study were distributed across the federal states of Germany. Two federal states did not participate in sampling. Both are city-states with only small hunting areas. One federal state took several times the required number of samples in wild boars and in roe deer. The impact of these additional samples on the overall prevalence estimates was considered minimal for the pathogens studied, as the prevalences recorded in this federal state were similar to those obtained without the inclusion of its samples (data not shown). The total number of samples from wild ducks and geese was low. Therefore, the obtained results should be interpreted with caution, and future studies including a higher number of samples, should be carried out to verify that the results obtained in this study can be extrapolated to the general population of wild ducks and geese in Germany.

As available studies have shown that the prevalence of bacteria and the results of the antimicrobial sensitivity analysis could be highly variable among different geographical locations (19), further analyses with respect to regional distribution and genetic traits need to be carried out to examine potential regional hot spots of AMR in wildlife in Germany.

Our results showed that even when *Salmonella* spp. were found in fecal samples from wild boars hunted in Germany, the prevalence is low. This is in accordance with previous reports from Spain, Portugal and Italy that likewise found low prevalence of *Salmonella* isolates from wild boar feces (52–55). However, substantially higher *Salmonella* prevalences have been be found in serum samples, tonsils or lymph nodes (31, 54, 55), or in animals co-habiting with livestock (56). *Salmonella* Enteritidis was the most frequent serotype, which agrees with previous investigations, which also detected *Salmonella* Enteritidis in wild boars (57). However, serovar *Salmonella* Choleraesuis that has been found increasingly in recent years in diseased wild boars in Germany (34, 35) was not detected in our study. A greater diversity of serotypes was recognized in Spain by Navarro-Gonzalez et al. (56) and Gil Molino et al. (55).

As Navarro-Gonzalez et al. (56), we found low resistance rates in the *Salmonella* isolates submitted for testing, with the vast majority of the isolates from wild boars being sensitive to all substances. This differs from previous studies that found higher resistance patterns with almost all isolates resistant to at least one antimicrobial substance (31, 35, 55). Despite the high proportion of fully susceptible isolates found in our study, resistance to ciprofloxacin and colistin were found in one *Salmonella* Enteritidis isolate each in agreement with previous studies (31).

The absence of *Salmonella* spp. in wild ducks and geese is in agreement with previous studies, where predominantly negative results or very low prevalence of *Salmonella* spp. in wild birds has been observed (27, 58–61). Therefore, as other authors hypothesized, the importance of wild birds in spreading *Salmonella* could be limited to those residing in areas that are highly contaminated by human waste or domestic animal manure (60, 61).

In our study, *Campylobacter* spp. were rarely found in roe deer feces. This is consistent with previous studies that suggest that wild cervids, and in particular roe deer, are of limited importance as *Campylobacter* reservoirs (28, 62–64). Although several authors have isolated *Campylobacter* spp. from wild deer, the number of studies that include their resistance profiles is still very limited. Carbonero et al. (65) reported more than 60% of the isolates from roe deer resistant to at least one antimicrobial substance, including streptomycin, tetracycline and ciprofloxacin. In our study, the two *Campylobacter jejuni* submitted to the BfR were susceptible to all tested substances.

Despite the fact that the intestinal tract of wild birds is considered a favorable environment for *Campylobacter* colonization, with reported prevalence ranging from 9.2 to 52.2% in wild ducks and geese (66, 67), *Campylobacter* spp. were absent in fecal samples from wild ducks and geese analyzed in this study. This absence could be due to loss of *Campylobacter* survival due to extreme temperatures, low water content, or ultra-violet light levels to which fecal content of bird cadavers sampled in this study were subjected.

Prevalence of STEC reported in wildlife in Europe shows a general pattern with a lower prevalence in wild boars (4.8–9%) than in deer (25–42%) (68–72). This is in line with our results. The isolates from wild boars and roe deer submitted to the BfR showed considerable diversity. The most prevalent Shiga toxin gene was *stx2*, whereas *stx1* was detected only in 40 isolates from roe deer and 5 from wild boars. This is also in concordance with previous studies carried out in Europe, which reported higher prevalence of *stx2* than of *stx1* among STEC isolates from wild ungulates (64, 69, 70, 72, 73). Our data reinforce the role of certain wild species as reservoirs of STEC strains that are potentially pathogenic to humans, as two isolates found in wild boars were described as *E. coli* O157:H7 (0.37%). Although there are studies in which this STEC serotype was absent in wild ungulates (28), in other studies prevalences of 0.75–3.41% are described (74, 75). Other clinical relevant serotypes (e.g., O103:H2 and O26:H11) with high similarity to human strains are also described in game meat in Germany (76). The serotype O27:H30, that has been associated with deer previously (71, 77), was found in three isolates from roe deer. Of the 150 STEC isolates analyzed at the BfR only one from each animal species showed resistance. The resistant isolate from wild boars showed resistance to six substance classes, including the (fluoro-)quinolones nalidixic acid and ciprofloxacin. This high percentage of isolates susceptible to the antimicrobial substances observed among STEC strains from wild animals has also been found in previous studies (71, 74).

Despite the fact that some studies suggest that wild birds could act as carriers of STEC, in general zero or low levels of STEC have been described in wild birds (62, 78, 79). This is in line with our findings.

As part of the physiological gut microbiota, commensal *E. coli* have been reported in wild mammals with high prevalence (52, 80, 81). Likewise, *E. coli* were found in our study in almost all the analyzed samples from wild boars and roe deer, but only in 50% (51 of 102) of the samples from wild ducks and geese. This observation is in the range described by previous studies that revealed a large variation in the prevalence of *E. coli* in geese, ranging from below 10–100% (59, 70, 82).

Resistance of commensal *E. coli* from wild animal fecal samples analyzed in this study were typically low. This is in agreement with the available literature, which shows in general low antimicrobial resistance rates among *E. coli* from wild ungulates (28, 80, 83–85) or wild birds (86), compared to livestock animals. To some concern, some isolates from the animal species included in this study exhibited resistance to 3rd generation cephalosporins (cefotaxime and ceftazidime), fluoroquinolones or colistin (in 1.8 and 2% of the isolates in wild boar and wild ducks and geese, respectively). Resistance to fluoroquinolones in wild ungulates has been previously described (81). Colistin resistance genes have been previously found in *E. coli* isolates from wild birds (87, 88), but to the best of our knowledge, this is the first report of colistin resistance in *E. coli* isolates from wild boars.

Our results showed that in Germany wild boars, roe deer and wild ducks and geese are reservoirs of ESBL-/AmpC-producing *E. coli*, which may reflect the general distribution of such bacteria in the environment outside of farm animal husbandry. Indeed, the proportion of positive samples found in wild boars corresponded roughly to the detection rate that was observed in a cross-sectional study in humans in Germany (89). The presence of ESBL-/AmpC-producing *E. coli* in wild animals is in line with previous studies on wild birds (33, 90–93) and wild ungulates (80, 84, 94, 95), which reported prevalences similar to those reported in our study.

Phenotypically grouped in ESBL-producers, AmpC-producers or ESBL+AmpC-producers, the ESBL-producing isolates dominated in all animal species included in this study, which might be linked with contact to human or livestock waste. The proportion of the AmpC- phenotype was higher in the isolates from roe deer (25%). High proportions and modest genetic diversity of ESBLs producing *E. coli* from wild animals have been previously reported (33, 91–93, 96).

Genotypically, CTX-M-1 was the most prevalent ESBL (51%), but in 36% of the isolates harbored a CTX-M-15 or CTX-M-9-group betalactamase. In livestock, CTX-M-1 is the most prevalent ESBL, especially in pigs and cattle, whereas CTX-M-15 and CTX-M-14 are detected only in minor proportions in livestock or meat (97, 98). One the other hand, in humans CTX-M-15 is predominant from clinical ESBL associated infection (99). Nevertheless, in non-clinical settings, CTX-M-1 is also found as the most prevalent ESBL variant (100). Therefore, a clear transmission route can not be derived from these data. Conceivable transmission could be manure fertilized fields, contaminated water sources or waste. Although SHV and CMY-2 is frequently detected in poultry production (101), none of these betalactamases were detected in ducks and geese and only one CMY-2-like isolate was found in wild boars. This might hint to hardly transmission from poultry production into the wild.

Among the confirmed ESBL-/AmpC-producing *E. coli* isolates from wild animals with characteristic resistance to betalactams, a significant percentage presented further resistance to up to five classes of substances, including fluoroquinolones. This percentage was numerically higher in wild ducks and geese, followed by wild boars and finally roe deer. In contrast to the non-selectively isolated commensal *E. coli*, resistance to colistin was not observed in ESBL-/AmpC-producing *E. coli*.

MRSA has been previously found in meat from wild boars in Germany (102). However, in our study it was noticeable that all isolates from wild boars sent to the BfR with suspicion of MRSA were not confirmed as MRSA, but instead turned out to be methicillin-susceptible *Staphylococcus aureus*. It could be assumed that the *S. aureus*, incorrectly identified as MRSA, were able to survive in the selective media because of other resistance mechanisms, such as increased beta-lactamase activity (103). The absence of MRSA in wild boars is in line with previous studies where MRSA were absent or rarely found in nasal swabs taken from wild boars (104–109).

Despite the low levels of resistance found in the animal species studied, our results underline that antimicrobial resistance is less frequent in roe deer, followed by wild boars and finally wild ducks and geese. This can be clearly seen in the resistance profiles of commensal *E. coli* and ESBL-/AmpC-producing *E. coli* (Figure 1), where data is available for all three studied animal species. This is in line with available data that suggest that carnivorous and omnivorous species are generally at a higher risk of AMR carriage (16). Particularly low resistance rates have been found in isolates from roe deer, which could demonstrate a lower level of exposure of roe deer to human and animal waste. Wild boars have been reported to carry resistant bacteria to a greater extent than other wild animal species, as in addition to their omnivorous behavior, their increased mobility and their high tolerance to human disturbance (19, 30), brings them to a closer contact with humans and livestock. On the other hand, the higher resistance found in wild ducks and geese might be attributable to a greater contact with wastewater or domestic animal manure containing high levels of bacteria carrying antibiotic resistance. However, we have to take into account that the low number of samples from wild ducks and geese analyzed in this study makes our margin of error larger, as shown by the wide confidence interval, so the actual prevalence of the population may vary. Future studies focusing simultaneously on several animal species living in the same habitat are needed to confirm the observed differences and determine the influencing factors.

One of the major concerns regarding the presence of resistant bacteria in wild animals is the potential contamination of meat with resistant bacteria during game meat production (110). Injuries to the digestive tract caused by gunshots, lower degree of bleeding compared to slaughtered animals and delayed evisceration of game bodies under suboptimal environmental conditions (111, 112), are the main factors that could contribute to such contamination. Since consumer exposure to resistant bacteria is possible through the consumption of contaminated meat (113), careful hygiene practices must be observed during harvesting, processing and marketing of game meat. Special attention should be paid to the presence of bacteria resistant to 3rd generation cephalosporins, fluoroquinolones, colistin or even carbapenems, which pose a serious public health concern. Further studies evaluating the relationship between the prevalence of resistance in feces from wild animals and the presence of resistant bacteria in game meat is needed in Germany in order to evaluate this potential pathway for human exposure to resistant bacteria.

The population size of the wild animals contemplated in this study has been increasing during the last decades in most of the European countries (18). This fact together with the increased fragmentation of natural ecosystems, has led to a greater proximity of these animal species to urban and peri-urban areas. Therefore, it may be advisable to investigate continuously the occurrence of resistant bacteria in wildlife. Additionally, as humans, livestock and environment play a relevant role in the origin of AMR in wild animals, a “One Health” approach would be essential when approaching it (114). Through this approach, efforts should focus on the determination of the role of wildlife in the dynamics of AMR, especially for those resistance traits to high priority substances for human and animal health. As the interpretation of resistance patterns also depends on the sampling techniques, the methodology and laboratory techniques employed to determine the susceptibility to antibiotics, standardization and harmonization need further improvement (19, 115) to allow for the comparison of data on AMR in wildlife between countries. Detailed regional studies will be required to identify factors affecting AMR in wild animals as well as potential pathways from which wildlife is acquiring resistant bacteria. In addition, the identification and evaluation of strategies to reduce the spread of AMR from humans and livestock to the environment and wildlife will be essential (116).

## Conclusions

Wild boars, roe deer and wild ducks and geese can be used as bioindicators or sentinels for the presence of resistant bacteria in the environment. Our results indicate that overall, the prevalence of resistant bacteria in the selected wild animals in Germany is low, which may reflect the low level of exposure of these animals to antimicrobials and the low level of resistant bacteria in the environment. However, the patterns observed in bacteria from the wild animals included in this study are an indicator for specific resistance traits in the environment, including those to highest priority substances such as 3rd generation cephalosporins, fluoroquinolones and colistin. To account for the low prevalence of AMR in wildlife in conjunction with the presence of resistance to critically important antimicrobials use of selective isolation in the continuous monitoring of the AMR in wildlife is advisable. Furthermore, the possible role of wildlife as reservoir of resistant bacteria would need to be assessed, as wild animals, and in particular wild ducks and geese could become spreaders of resistant bacteria given their capacity for long-range movements.

## Data Availability Statement

The data analyzed in this study is subject to the following licenses/restrictions: The dataset used in this article belongs to the German National Zoonoses Monitoring Program. Currently these data are not publicly available, however work is currently underway to create a public database that contains this data along with many other data from other programs and years. Requests to access these datasets should be directed to Carolina Plaza-Rodríguez, Carolina.Plaza-Rodriguez@bfr.bund.de.

## Author Contributions

Conceptualization: CP-R and B-AT. Formal analysis: CP-R, B-AT, and KA. Original draft preparation: CP-R. Review and editing: CP-R, KA, MG, JAH, AI, IS, KS, ES, LW, BP, SN, AK, and B-AT. All authors contributed to the article and approved the submitted version.

## Conflict of Interest

The authors declare that the research was conducted in the absence of any commercial or financial relationships that could be construed as a potential conflict of interest.
