# Supplementary material for: Wildlife as Sentinels of Antimicrobial Resistance in Germany?
Source: Front Vet Sci. 2021 Jan 27;7:627821. doi: 10.3389/fvets.2020.627821 (PMC7873465; doi:10.3389/fvets.2020.627821)
Supplement: Supplementary file 1 [file Table_1.DOCX]

**Supplement 1:** Resistance testing of *Salmonella* spp. and *E. coli.* Overview of the active ingredients used, the tested concentration ranges, as well as the evaluation criteria. The evaluation was carried out as far as possible based on the implementation decision 2013/652/EU.

| **Substances classes** | **Antimicrobial agents** | **Cut-off-Value ≤**  (µg/ml) | | **Concentration range**  (µg/ml) | |
| --- | --- | --- | --- | --- | --- |
|  |  | ***Salmonella* spp.** | ***E. coli*** | **Minimum** | **Maximum** |
| Aminoglycosides | Gentamicin | 2 | 2 | 0.25 | 32 |
| Amphenicols | Chloramphenicol | 16 | 16 | 2 | 64 |
| Cephalosporins | Cefotaxime | 0.5 | 0.25 | 0.06 | 4 |
|  | Ceftazidime | 2 | 0.5 | 0.25 | 16 |
| (Fluoro)quinolones | Nalidixic acid | 16 | 16 | 4 | 64 |
|  | Ciprofloxacin | 0.06 | 0.06 | 0.008 | 8 |
| Aminopenicillins | Ampicillin | 8 | 8 | 0.5 | 32 |
| Polymyxins | Colistin | 2 | 2 | 2 | 4 |
| Folic Acid Synthesis Inhibitors | Sulfamethoxazole | 256^a^ | 64 | 8 | 1024 |
|  | Trimethoprim | 2 | 2 | 0.5 | 32 |
| Tetracyclines | Tetracycline | 8 | 8 | 1 | 64 |
| Azalides | Azithromycin | 16^a^ | 16^a^ | 2 | 64 |
| Carbapenems | Meropenem | 0.125 | 0.125 | 0.03 | 16 |
| Glycylcyclines | Tigecycline | 1 | 1 | 0.25 | 8 |

^a^ Values not defined in Implementing Decision 2013/652 / EU. EFSA recommendation for uniform assessment within the EU.

**Supplement 2:** Resistance testing of *Campylobacter* (*C.*) *jejuni* and *C. coli.* Overview of the active substances used, the concentration ranges tested and the evaluation criteria. The evaluation was carried out taking into account the stipulations in the implementation decision 2013/652/EU.

| **Substances classes** | **Antimicrobial agents** | **Cut-off-Value ≤**  (µg/ml) | **Concentration range**  (µg/ml) | |
| --- | --- | --- | --- | --- |
|  |  |  | **Minimum** | **Maximum** |
| Aminoglycosides | Gentamicin | 2 | 0.125 | 16 |
|  | Streptomycin | 4 | 0.025 | 16 |
| (Fluoro)quinolones | Nalidixic acid | 16 | 1 | 64 |
|  | Ciprofloxacin | 0.5 | 0.125 | 16 |
| Tetracyclines | Tetracycline | 1*/2** | 0.5 | 64 |
| Macrolides | Erythromycin | 4*/8** | 1 | 128 |

* Cut-off values for *C. jejuni.*
** Cut-off values for *C. coli*.

**Supplement 3:** Overview of the active substances used for broth microdilution, the concentration ranges tested and the evaluation criteria. The evaluation was carried out taking into account the technical specifications proposed by EFSA.

| **Substances classes** | **Antimicrobial agents** | **Cut-off-Value ≤**  (µg/ml) | **Concentration range**  (µg/ml) | |
| --- | --- | --- | --- | --- |
|  |  |  | **Minimum** | **Maximum** |
| Aminoglycosides | Gentamicin | 2 | 1 | 16 |
|  | Kanamycin | 8 | 4 | 64 |
|  | Streptomycin | 16 | 4 | 32 |
| Amphenicols | Chloramphenicol | 16 | 4 | 64 |
| (Fluoro)quinolones | Ciprofloxacin | 1 | 0.25 | 8 |
| Penicillins | Penicillin G | 0.12 | 0.12 | 2 |
| Cephalosporins | Cefoxitin | 4 | 0,5 | 16 |
| Folic Acid Synthesis Inhibitors | Trimethoprim | 2 | 2 | 32 |
| Sulfonamides | Sulfamethoxazol | 128 | 64 | 512 |
| Tetracyclines | Tetracycline | 1 | 0.5 | 16 |
| Lincosamides | Clindamycin | 0.25 | 0.12 | 4 |
| Macrolides | Erythromycin | 1 | 0.25 | 8 |
| Pseudomonic acids | Mupirocin | 1 | 0.5 | 256 |
| Ansamycins | Rifampicin | 0.03 | 0.016 | 0.5 |
| Oxazolidinones | Linezolid | 4 | 1 | 8 |
| Triterpenic acids | Fusidic acid | 0.5 | 0.5 | 4 |
| Streptogramins | Quinupristin/Dalfopristin | 1 | 0.5 | 4 |
| Pleuromutilins | Tiamulin | 2 | 0.5 | 4 |
| Glycopeptides | Vancomycin | 2 | 1 | 16 |

**Supplement 4:** Results of the investigation of the STEC isolates from wild boar sent in for Shiga toxin including the Shiga toxin-coding genes (*stx1* and *stx2*) as well as the *eae* and *ehxA* genes.

| **O-Group** | **H-Group^1^** | ***stx1*-Gen** | ***stx2*-Gen** | **Shiga toxin** | ***eae*-Gen** | ***ehxA****-***Gen** | **N° Isolates** |
| --- | --- | --- | --- | --- | --- | --- | --- |
| 100 | [H30] | - | + | + | - | - | 1 |
| 110 | H45 | - | + | + | - | + | 1 |
| 128 | [H2] | + | + | + | - | + | 1 |
| 146 | H28 | - | + | + | - | - | 1 |
| 146 | [H21] | + | - | + | - | + | 1 |
| 146 | [H28] | - | + | + | - | + | 1 |
| 146 | [H28] | - | + | + | - | - | 3 |
| 15 | [H16] | - | + | - | - | - | 1 |
| 157 | H7 | + | + | + | + | + | 1 |
| 157 | H7 | - | + | + | + | + | 1 |
| 187 | [H28] | - | + | - | - | + | 1 |
| 23 | H8 | + | + | + | - | + | 2 |
| 26 | [H11] | - | + | + | + | + | 1 |
| 27 | [H30] | - | + | + | - | + | 1 |
| 27 | [H30] | - | + | + | - | - | 2 |
| 36 | H19 | - | + | + | - | - | 1 |
| 36 | [H14] | - | + | - | - | + | 1 |
| 45 | [H2] | - | + | + | + | + | 1 |
| 85 | [H18] | - | + | + | - | + | 1 |
| rough | [H30] | - | + | + | - | + | 1 |
| **TOTAL** |  | **5** | **23** | **21** | **4** | **15** | **24** |

**^1^** H-types in square brackets were determined by molecular methods.

**Supplement 5:** Results of the investigation of the STEC isolates from roe deer sent in for Shiga toxin including the Shiga toxin-coding genes (*stx1* and *stx2*) as well as the *eae* and *ehxA* genes.

| **O-Group** | **H-Group**^1^ | ***stx1*-Gen** | ***stx2*-Gen** | **Shiga toxin** | ***eae*-Gen** | ***ehxA*-Gen** | **N° Isolates** |
| --- | --- | --- | --- | --- | --- | --- | --- |
| 6 | [H49] | - | + | + | - | + | 1 |
| 6 | [H49] | - | + | + | - | - | 1 |
| 8 | [H19] | + | - | + | - | - | 2 |
| 11 | [H48] | + | - | + | - | - | 4 |
| 11 | [H48] | + | - | - | - | - | 1 |
| 11 | [H5] | - | + | + | - | + | 1 |
| 12 | H45 | + | - | - | - | - | 1 |
| 12 | [H45] | + | - | + | - | - | 1 |
| 12 | [H45] | + | - | - | - | - | 1 |
| 21 | H21 | - | + | + | - | + | 1 |
| 21 | [H21] | - | + | + | - | + | 5 |
| 21 | [H21] | - | + | + | - | - | 1 |
| 21 | [H21] | - | + | - | - | + | 1 |
| 26 | [H11] | + | - | + | + | + | 1 |
| 27 | H30 | - | + | + | - | - | 1 |
| 27 | [H30] | - | + | + | - | - | 2 |
| 36 | [H14] | - | + | - | - | + | 3 |
| 43 | [H2] | - | + | + | - | + | 4 |
| 43 | [H2] | - | + | + | - | - | 2 |
| 43 | [H2] | - | + | - | - | + | 1 |
| 79 | [H23] | + | - | - | - | - | 3 |
| 79 | [H23] | - | + | - | - | - | 1 |
| 88 | [H] | - | + | + | - | + | 1 |
| 110 | [H31] | + | - | + | - | + | 1 |
| 110 | [H31] | - | + | + | - | + | 2 |
| 128 | [H2] | + | + | + | - | + | 2 |
| 128 | [H2] | + | + | + | - | - | 1 |
| 128ac | [H2] | + | + | + | - | + | 1 |
| 128ac | [H2] | + | - | + | - | + | 1 |
| 146 | [H28] | + | + | + | - | + | 1 |
| 146 | [H28] | + | + | + | - | - | 1 |
| 146 | [H28] | - | + | + | - | + | 9 |
| 146 | [H28] | - | + | + | - | - | 28 |
| 146 | [H28] | - | + | - | - | - | 1 |
| 154 | [H31] | + | - | + | - | - | 3 |
| 154 | [H31] | + | - | - | - | - | 8 |
| 174 | [H8] | + | - | + | - | + | 1 |
| 187 | [H28] | - | + | + | - | + | 6 |
| 187 | [H28] | - | + | - | - | - | 1 |
| NT | [H21] | - | + | + | - | + | 1 |
| NT | [H23] | + | - | + | - | - | 1 |
| NT | [H28] | - | + | + | - | + | 1 |
| NT | [H28] | - | + | + | - | - | 1 |
| NT | [H31] | - | + | + | - | + | 1 |
| NT | [H31] | - | + | - | - | + | 1 |
| NT | [H45] | - | + | + | - | + | 1 |
| NT | [H4] | - | + | + | - | + | 1 |
| NT | [H7] | - | + | + | - | + | 1 |
| NT | [H8] | - | + | + | - | + | 1 |
| NT | [H] | + | - | + | - | - | 1 |
| rough | [H21] | - | + | + | - | + | 1 |
| rough | [H23] | + | - | + | - | - | 1 |
| rough | [H23] | + | - | - | - | - | 2 |
| rough | [H28] | - | + | + | - | + | 1 |
| rough | [H28] | - | + | + | - | - | 1 |
| rough | [H45] | + | - | + | - | - | 1 |
| rough | [H21] | - | + | + | - | + | 1 |
| **TOTAL** |  | **40** | **92** | **101** | **1** | **54** | **126** |

**^1^** H-types in square brackets were determined by molecular methods.
